# Supplementary material for: Functional ultrasound imaging and neuronal activity: how accurate is the spatiotemporal match?
Source: bioRxiv. 2024 Jul 10:2024.07.10.602912. Preprint. [Version 1] doi: 10.1101/2024.07.10.602912 (PMC11257620; doi:10.1101/2024.07.10.602912)
Supplement: Supplement 1 [file NIHPP2024.07.10.602912v1-supplement-1.pdf]

bioRxiv preprint doi: https://doi.org/10.1101/2024.07.10.602912; this version posted July 10, 2024. The copyright holder for this preprint (which was not certified by peer review) is the author/funder, who has granted bioRxiv a license to display the preprint in perpetuity. It is made available under aCC-BY 4.0 International license.

Supplementary Materials

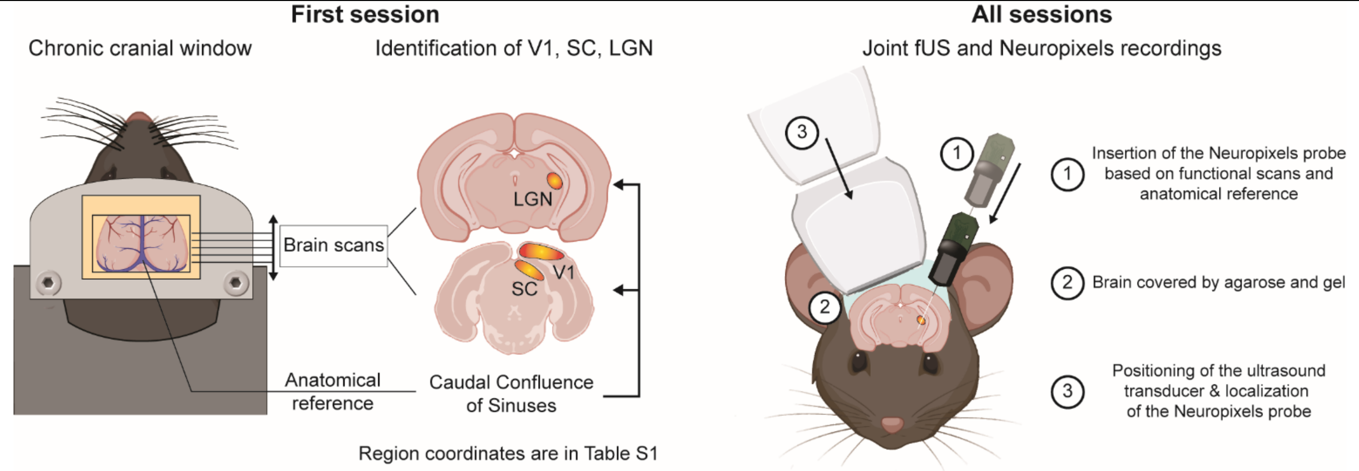

Figure S1. Schematic representation of the data acquisition procedure.

bioRxiv preprint doi: https://doi.org/10.1101/2024.07.10.602912; this version posted July 10, 2024. The copyright holder for this preprint (which was not certified by peer review) is the author/funder, who has granted bioRxiv a license to display the preprint in perpetuity. It is made available under aCC-BY 4.0 International license.

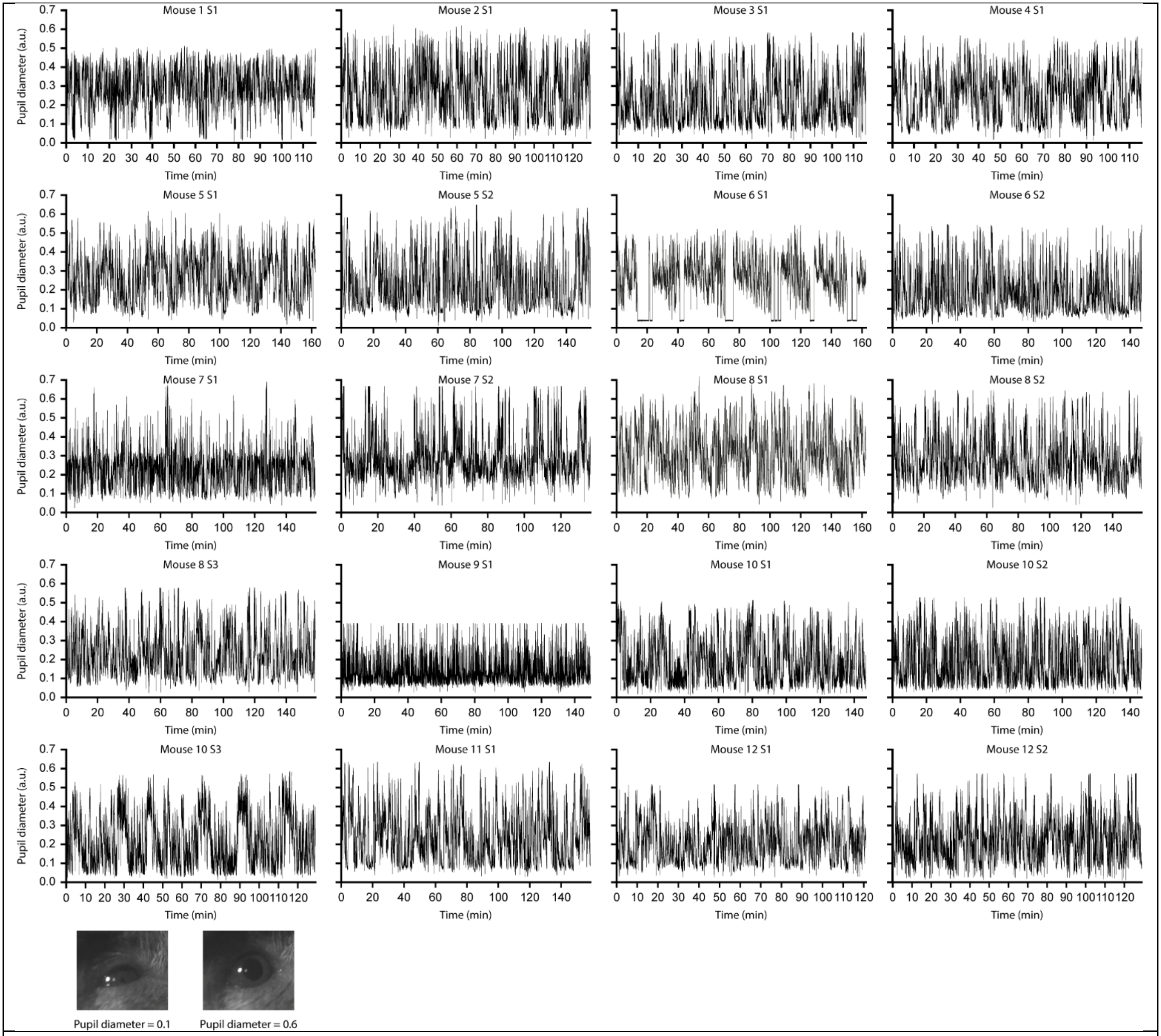

Figure S2. Pupil diameter variation across experimental sessions.
Top, Normalized variation of pupil diameter tracked across sessions confirming for alertness of mice along recordings. *Bottom,* Example video frames used to compute the mouse's pupil diameter with small (left) and large pupil size (right).

bioRxiv preprint doi: https://doi.org/10.1101/2024.07.10.602912; this version posted July 10, 2024. The copyright holder for this preprint (which was not certified by peer review) is the author/funder, who has granted bioRxiv a license to display the preprint in perpetuity. It is made available under aCC-BY 4.0 International license.

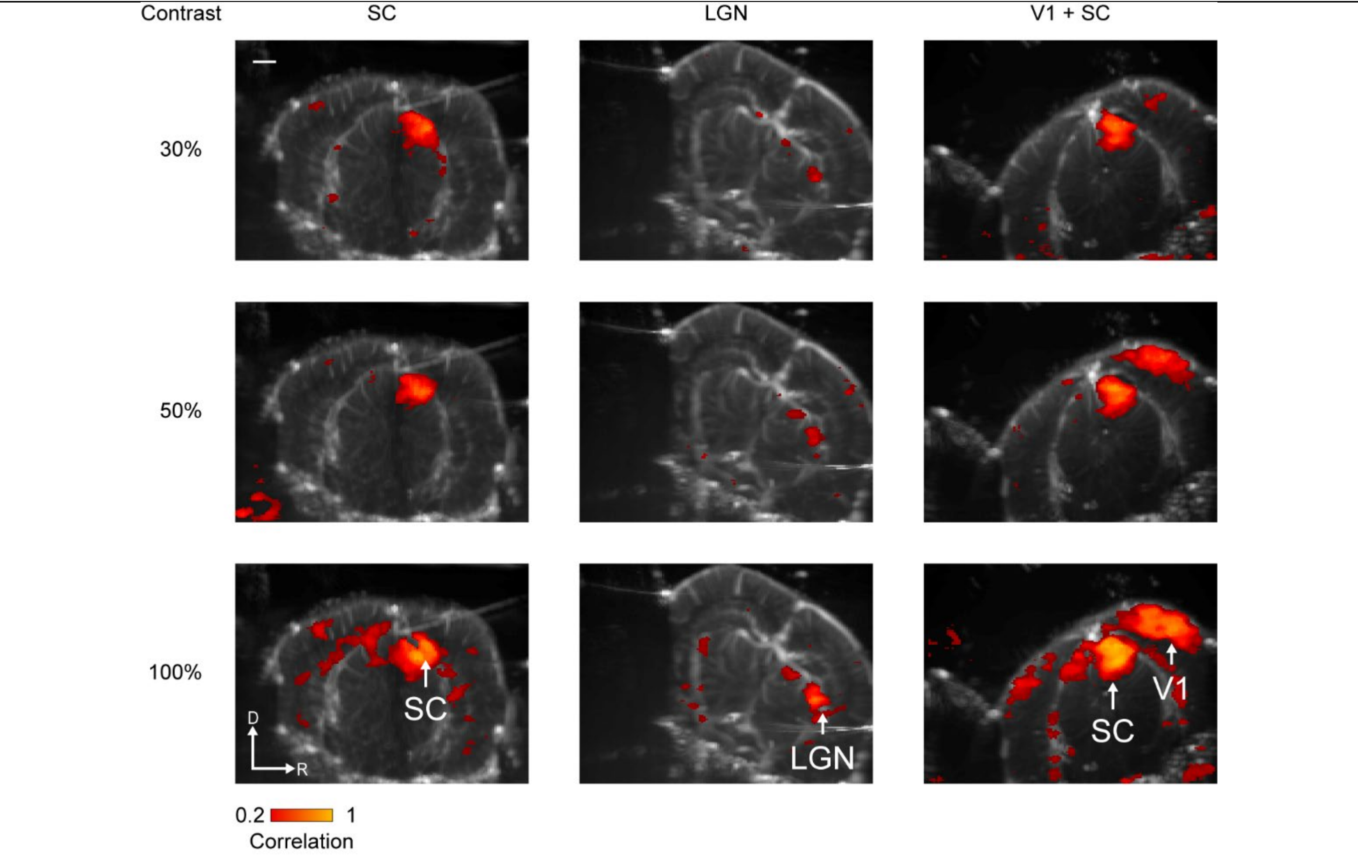

Figure S3. Correlation maps across regions and sessions.
Example of correlation maps generated by computing the correlation between fUS voxels and the stimulation pattern across contrast conditions (50 trials each) and regions of interest (one session per region). Scale bar: 1 mm.

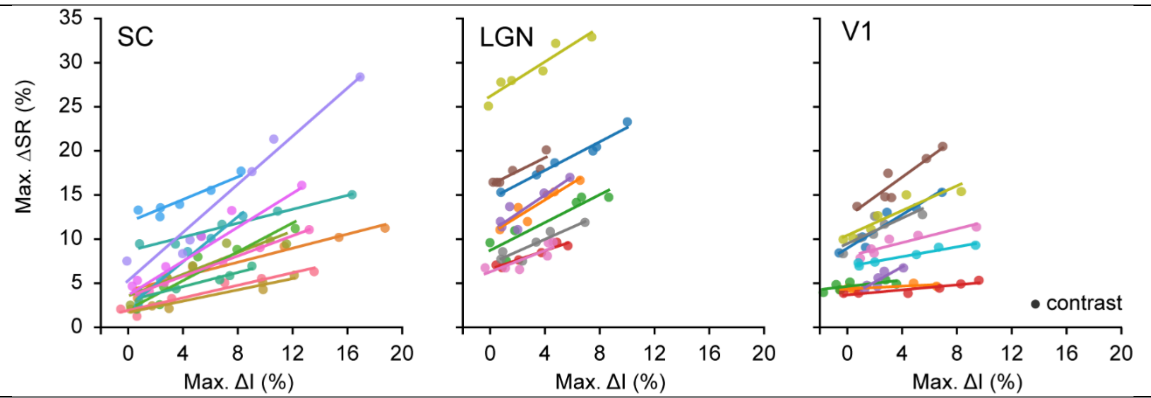

Figure S4. fUS and spike rate activities in responses to contrast conditions.
Non-normalized maximum of spike rate variation (max. ΔSR in %) with respect to the maximum of fUS signal variation (max. ΔI in %). Color corresponds to a recording session and dot represents a contrast condition.

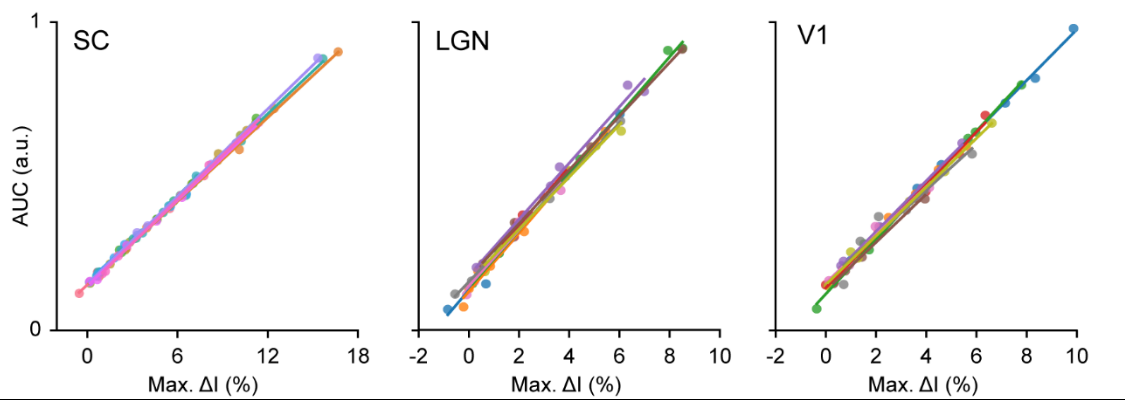

bioRxiv preprint doi: https://doi.org/10.1101/2024.07.10.602912; this version posted July 10, 2024. The copyright holder for this preprint (which was not certified by peer review) is the author/funder, who has granted bioRxiv a license to display the preprint in perpetuity. It is made available under aCC-BY 4.0 International license.

Figure S5. Correlation between maximum ∆I and area under the curve.
Maximum of fUS signal variation (max. ∆I in %) with respect to the area under the curve of fUS signal variation (AUC in arbitrary units a.u.). Color corresponds to a recording session and dot represents a contrast condition.

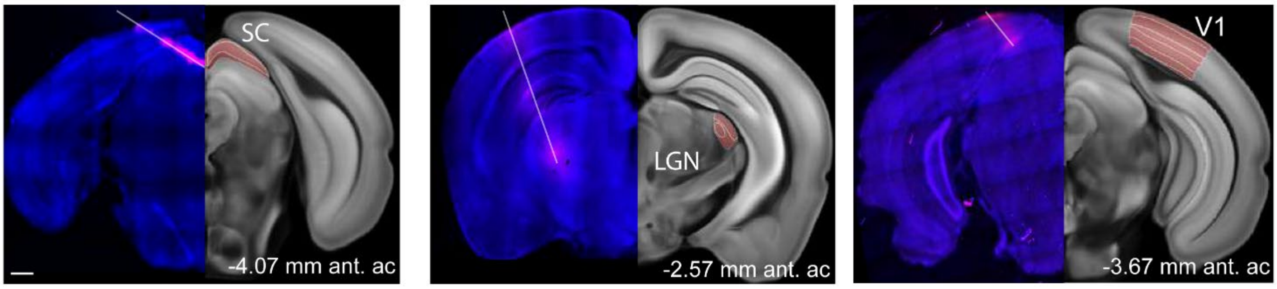

Figure S6. Neuropixels probe trajectories through the mouse brain.
Histological reconstruction of the Neuropixels probe trajectories (white lines) showing DAPI staining (blue) and the fluorescent indicator DiI (pink) used to coat the probe. Trajectories are in the superior colliculus, lateral geniculate nucleus, or primary visual area; from left to right. Scale bar: 0.6mm.

Table S1. Mice and sessions.

**Mouse ID**	**Number of sessions per region**		
	**SC**	**LGN**	**V1**
1	2		
2	1		
3	2		
4	1		
5	1	2	
6	1	1	1
7	1	1	2
8	3	1	3
9		1	
10		2	1
11		1	
12			2
**Total**	12	9	9

Table S2. Coordinates used for Neuropixels probe insertion. CCS: Caudal confluence of Sinuses.

**Regions of interest**	**Position relative to CCS (mm)**	**Lateral position from midline (mm)**	**Angle (relative to horizontal)**	**Insertion depth (mm)**
**SC**	+0.5	2.5	25°	3.0
**V1**	+0.4	3.0	12°	3.5
**LGN**	+1.6	3.6	60°	3.5
